# Assessment of the axilla in women with early-stage breast cancer undergoing primary surgery: a review

**DOI:** 10.1186/s12957-024-03394-6

**Published:** 2024-05-09

**Authors:** Justin James, Michael Law, Shomik Sengupta, Christobel Saunders

**Affiliations:** 1 Eastern Health, Melbourne, Australia; 2 Monash University, Melbourne, Australia; 3 University of Melbourne, Melbourne, Australia; 4 Department of Breast and Endocrine Surgery, Maroondah Hospital, Davey Drive, Ringwood East, Melbourne, VIC 3135 Australia

## Abstract

Sentinel node biopsy (SNB) is routinely performed in people with node-negative early breast cancer to assess the axilla. SNB has no proven therapeutic benefit. Nodal status information obtained from SNB helps in prognostication and can influence adjuvant systemic and locoregional treatment choices. However, the redundancy of the nodal status information is becoming increasingly apparent. The accuracy of radiological assessment of the axilla, combined with the strong influence of tumour biology on systemic and locoregional therapy requirements, has prompted many to consider alternative options for SNB. SNB contributes significantly to decreased quality of life in early breast cancer patients. Substantial improvements in workflow and cost could accrue by removing SNB from early breast cancer treatment. We review the current viewpoints and ideas for alternative options for assessing and managing a clinically negative axilla in patients with early breast cancer (EBC). Omitting SNB in selected cases or replacing SNB with a non-invasive predictive model appear to be viable options based on current literature.

## Introduction

Breast cancer was the most common cancer in Australia in 2020, with 18,909 new cases [1]. 85% of these were diagnosed at stage 1 or 2, where surgical management plays a central role. Ideally, surgery should treat the breast and axilla in a single-stage operation. It should give the best chance of breast preservation, cosmesis and maintenance of quality of life without compromising cure. Surgical management of the axilla has evolved from radical axillary dissection to relatively simpler SNB.

## Current methods of axilla management

### Axillary lymph node dissection (ALND)

#### ALND in node positive EBC

Traditionally, ipsilateral ALND has been integral to breast cancer surgery. Halsted’s paradigm of the stepwise progression of the disease formed the basis of this approach [2]. ALND is considered the standard of care in people with clinically node-positive breast cancer undergoing primary surgery, though its role is not proven by level-one evidence. Increasingly, clinically node-positive disease will be treated with neoadjuvant systemic treatment in modern practice. Limiting axillary surgery to SNB and targeted axillary dissection is now acceptable once nodal disease responds completely, clinically and radiologically.

#### ALND in node negative EBC

ALND is no longer the standard of care in clinically node-negative EBC. It is widely agreed that no clear evidence exists to prove that axillary dissection imparts a survival advantage in node-negative disease [3–5]. However, it may be important for locoregional control. Axelsson et al., based on “The Danish Breast Cancer Cooperative Group” register, report a better axillary recurrence-free survival in node-negative low-risk patients when ten or more nodes are removed by axillary dissection [6]. The NSABP B- 04 trial concludes that half of all patients with clinically occult axillary nodal metastasis will develop clinically relevant axillary recurrence without axillary dissection. These findings support the view that ALND helps to improve regional control [3, 4, 7–10]. However, this improvement in regional control does not improve overall survival [3]. 

Although the therapeutic role of axillary dissection has been challenged, axillary node status is still one of the strongest independent predictors of disease-free and overall survival [11–13]. ALND accurately identifies nodal metastasis if at least ten lymph nodes are removed. ALND then provides crucial prognostic information [14–17]. Extent of nodal disease is related to outcome. Although 83% of node-negtive patients survive five years, microscopic metastasis in the lymph node alone causes a significant reduction in the disease-free interval [18, 19]. Only 28% of women survive long-term when more than 13 nodes are positive [20]. Extra-nodal extension is also a marker of poor prognosis [21]. These pieces of information form the basis for selecting appropriate adjuvant treatment.

### SNB

SNB was introduced as a less invasive targeted option to obtain the required prognostic information and has become the standard of care in patients with clinically and radiologically node-negative early breast cancer. Krag et al. described the sentinel node technique (first in melanoma and later in breast cancers), and Giuliano and many others later validated it in node-negative breast cancers [22, 23]. Sentinel nodes can be identified in more than 95% of cases with a false negative rate of less than 6–10% and overall accuracy of over 96% [24–29]. Prognostic estimation and treatment decisions can be accurately made based on sentinel node status as it reflects the true axillary status [23, 30]. The axillary recurrence rate is less than 1% after a negative SNB.

## Arguments for improvement of current methods of axilla management

The adverse effects of ALND are substantial. Lymphedema (14%), limited shoulder/ arm motion (28%), and neuropathic pain (31%) are some of the main consequences of ALND [31]. 

Several shortcomings of SNB-based management of the axilla have been noted. The morbidity of SNB, even though better than axillary dissection, is still substantial. Up to 7% of patients suffer arm pain and swelling at the end of 2 years. Lymphoedema, intercostal brachial neuralgia, and shoulder stiffness are seen among many patients after SNB [32–36]. All these symptoms result in a substantial reduction in the quality of life in many cancer survivors [37]. Allergic reactions to blue dye often used to detect the sentinel node occur in 0.7% of all SNB procedures [38]. The accuracy of SNB depends on the number of lymph nodes examined. It is estimated that four lymph nodes may be optimal [39]. NSABP B32 suggested that at least two nodes are necessary to reduce the false-negative rate [38]. Removing just one sentinel lymph node may be associated with a higher recurrence rate [40, 41]. Failure in identifying the sentinel node occurs in 2% of surgeries [42]. 

Mammographic screening has resulted in a shift in the stage of breast cancer diagnosis [43]. Screen-detected cancers will have a lower chance of node positivity [44]. Two-thirds of patients undergoing SNB will have a negative result and hence be considered subject to unnecessary overtreatment as there is no benefit in removing uninvolved axillary nodes [38, 45–47]. 

Even when the sentinel node is found to be positive, further axillary surgery is no longer mandatory. More than 60% of patients with SNB-positive disease will have no further disease in the axilla and, therefore, will be subjected to over-treatment if the policy of completion axillary dissection is followed [25, 48, 49]. In patients with early breast cancer undergoing breast-conserving surgery, a finding of up to two positive sentinel nodes does not need to be automatically followed by axillary dissection [50, 51]. Similarly, axillary radiation is adequate for mastectomy patients to treat a positive axilla [52, 53]. Four studies found no survival advantage in performing axillary dissection in this group [54–57]. Similar to the low regional recurrence rates seen in the undissected axilla after negative SNB, those with untreated axilla but positive SNB also show satisfactory regional control [46, 58]. Current evidence favours no axillary dissection in clinically node-negative, SNB-positive early breast cancer [59, 60]. Systemic therapy is usually recommended for SNB-positive patients, and these treatments are not often altered by the number of nodes (except in selected phenotypes and clinical scenarios), further negating the need for completion ALND [61]. ALND may be omitted if the risk of nodal metastasis is less than 5% [62]. No further axillary treatment is needed, even if one or more micrometastases are detected in the SNB [57]. Various studies of patterns of care have demonstrated a decline in the rate of axillary dissection in the last decade, even when underlying trial criteria are not fulfilled [63]. Certainly, in selected circumstances (premenopausal women, those with cancers of non-luminal phenotype), the information from SNB or subsequent ALND may be critical; however, in a large number of EBCs, this may be found redundant.

From this information, we can conclude that a positive SNB leads to many decision-making challenges and potentially substantial overtreatment. A positive SNB is often a moment of anguish in a patient’s breast cancer journey. Further axillary surgery following SNB causes the emotional and physical trauma of a second operation. It leads to delay in the commencement of adjuvant treatment and increased complications and long-term sequelae [64]. 

Systemic therapy is not entirely decided by SNB status [62, 65–67]. Biological properties of the primary tumour dictate the type of systemic treatment. The risk of recurrence predicted by the SNB status is becoming less critical in this decision-making [68]. In fact, the INSEMA trial estimated that 99% of all patients enrolled in the trial (T1-2 CN0 cancers) could have their systemic treatment planned without the need for SNB [8]. If adjuvant management decisions are made regardless of the nodal status, SNB is unnecessary [69, 70]. There is also increasing evidence that axillary treatment or assessment is unnecessary in early EBC in elderly patients [4, 9, 71, 72]. Redundancy of the information gained by the SNB procedure has prompted many investigators to look for alternative methods of axillary management in EBC.

NSABP 04, Z0011 and IBSCG23-01 trials showed us the same findings: leaving behind clinically occult lymph node metastasis does not significantly increase regional recurrence or decrease survival [37]. A non-invasive alternative to SLNB, which will predict node negativity with sufficient accuracy and give similar prognostic information, will be most desirable in such low-risk cases [73]. A reliable, non-invasive estimate of axillary tumour burden could replace SNB, as ALND is not recommended in cases with less than two positive axillary nodes [74]. A modest estimate is that every second patient with early breast cancer could benefit from even a selective omission of SNB [8]. Cost savings and workflow efficiency improvements accompanying the omission of SNB are also substantial.

Alternative strategies for axillary management:

Many Alternative options for axillary management are reported in the literature. (Table 1)

**Table 1:** Alternative strategies for the management of clinically negative axilla in Early Breast Cancer

No SNB
SOUND Trial [71]
BOOG 213-08 [37]
INSEMA [8]
Tucker et.al [75]
Choosing wisely [76]
Radiological assessment
USS
MRI
PET scan
Predictive models
Clinical
Radiomics
Molecular characteristics of the tumour
Combined

### Selective omission of axillary assessment

Four randomised controlled trials are underway that look at omitting SNB in selected groups of patients. (Table 2) While the SOUND trial only includes patients with tumours less than 2 centimetres, the other three include both T1 and T2 tumours. These studies will examine the effectiveness of managing EBC without SNB in selected groups of patients. At a median follow-up of 5.7 years, the SOUND trial has found the omission of SNB is not inferior in terms of distant disease-free survival to SNB [77]. The Choosing Wisely recommendation developed by the Society of Surgical Oncology and subsequent ASCO guidelines on the management of axilla recommends that SNB is not required routinely in hormone-positive early breast cancer in women over 70 years of age.

**Table 2:** Strategies for the selective omission of SNB

Recommendations/ study	Group selected for management without SNB
SOUND Trial [71]	T1, any age, BCS with RT, Negative axilla USS
BOOG 213-08 [37]	T1-2, age > 18, BCS with RT, Negative axilla USS
INSEMA [8]	T1-2, age > 18, BCS with RT, c/I N0
Tucker et.al [75]	T1-2, age > 18, BCS with RT, Negative axilla USS
Choosing Wisely [76]	> 70 years old with hormone-positive, HER2 negative, T1N0

BCS Breast-conserving surgery, RT Radiotherapy

### Radiological evaluation of axilla

Dedicated radiological evaluation of the axilla using ultrasound scans (USS), Magnetic resonance Imaging (MRI), and Positron Emission Tomography (PET) has been attempted. Axillary USS is very specific but lacks the negative predictive value required to replace SNB [78, 79]. Many modifications of axillary USS have been suggested with improvements in reported results. Preoperative localisation of the sentinel node using contrast-enhanced USS (CEUS) and biopsy of suspicious nodes has been attempted as one of the strategies to replace SNB. A systematic review showed a pooled sensitivity of 54% and specificity of 100% in identifying positive axillary nodes by this technique [80]. Adding shear wave elastography to conventional ultrasound improves the accuracy but is still inferior to the CEUS results [81]. A dedicated MRI assessment may have the required negative predictive value and sensitivity. However, it has yet to be validated in large studies [82]. PET (using FDG PET/CT) has also shown a high specificity. However, it is widely agreed that better sensitivity and diagnostic performance are needed to adopt this test as an alternative to SNB [83]. Radiological assessment of the axilla with all these advanced imaging techniques appears promising but is not a viable stand-alone alternative to SNB.

### Prediction models

#### Clinicopathological models

Non-invasive estimates of axillary nodal status based on characteristics of the primary lesion (clinical, radiological, histopathological, and molecular) and the appearance of the axilla (clinical and radiological) have been attempted by many researchers. One such method is a nomogram developed by Bevilacqua et al. from the Memorial Sloan Kettering Cancer Centre, incorporating nine commonly estimated clinicopathological parameters of the primary tumour to predict the chance of sentinel node positivity in early breast cancer patients [84]. Sentinel node status can be predicted with an accuracy of 73% uing this nomogram. Similar prediction models for sentinel node status based on the clinicopathological and radiological parameters have been reported by many others [12, 36, 62, 65, 73, 84–103]. Predictive models for sentinel node status estimation incorporating various combinations of preoperative features are summarised in Table 3. Patani et al. in 2007 reviewed available literature on predictive factors of axillary disease [104] and concluded that these methods have yet to be found reliable enough to replace formal assessment of the axilla by SNB. Table 4 summarises the most commonly used and validated clinicopathological models and their reported accuracy. Overall, all these models have similar accuracy.

**Table 3:** Predictive models for Sentinel node status

Model	Clinical characteristics						Histological characteristics					Molecular characteristics							USS features of lymph nodes		
	Age	Menopausal status	Race	Location	Palpable primary	T	Histological type	LVI	Grade	Multifocality	Margins	ER	PR	Her2	Ki67	Nm-23	Kiss-1	S phase	Transverse diameter	Cortical thickness	Hilum
Bevilacqua	+			+		+	+	+	+	+		+	+								
Carmichael	+			+	+	+	-	+	+												
Chen K###	+			+		+	+	+	+			-	-	+							
Chen JY (Shanghai Model)	+			+		+	+	+													
Chadha##	-					+		+	-									-			
Ahlgren#						+															
Laurentiis++++						+	+	+		+											
Barth+++					+	+		+	+												
Choong++	+					+		+	+												
Mustafa	+					+			+												
Reyal+	+					+		+				+		+							
Kolarik ****	+			+		+	+	+	+	+		+	+	+	+						
Xie F		+				+						+	+	+		+	+				
Fehm T													+	+	+						
Qiu SQ						+			+			+							+	+	+
Ravdin	+					+							+					+			
Silverstein					+	+		+	+												
Takada*	+					+			+					+					+		+
Olivotto	+				+	+	+	+			+										
Gann	+	+		+		+	+		+			+	+					+			
Chua ***					+	+		+		+											
Viale						+	+	+		+			+								
Chagpar **	+				+	+		+													
Greer		+				+		+													
Chen W	+			+		+	+	+	+												

*AI-based model using Alternating Decision Tree (AD Tree).

** Two separate models: one with LVI and one without LVI; palpable tumour is a predictor when LVI is not included.

*** In clinically node-negative patients, the risk factors were palpability of the primary tumour, grade, LVI, and multifocality.

**** Preoperative and postoperative models. The postoperative model is accurate, while the preoperative model is not. In addition to the factors listed, the model also included the hospital operated and BMI.

+ Molecular subtypes approximated using ER, and PR is one of the factors.

++ Used a special statistical method- “MEE”; and instead of nuclear grade, nuclear pleomorphism was used.

+++Model specifically looks at T1 tumours.

++++ Tumour type is classified into three based on grade and histological subtypes. Irregular borders and nipple inversion are two other specific factors in this model (altogether, six factors in the model).

# Tumour size and clinically palpable node are the only factors. SPF or ER, PR status did not contribute.

## Model for T1 Tumours.

### Separate models for prediction of node positivity and pN2-3 status.

*T Primary tumour size, ER Oestrogen receptor, PR Progesterone receptor, HER2 Human Epidermal Growth Factor 2, AI Artificial intelligence, LVI Lymphovascular invasion, MEE maximum entropy estimation, SPF S phase fraction*

**Table 4:** Validated clinicopathological models in use and their accuracy

Model	Variables	Reported AU ROC	Ext validation
MSKCC	Age, T, LVI, location, multifocality, histologic type and grade, ER and PR	0.754	Yes
MDACC [112]	Age, T, LVI, location, multifocality, histologic type, Triple negative status	0.808	Yes
Paris	Age, T, LVI, Subtype	0.72	Yes
Shanghai	Age, T, LVI, location, histologic type	0.758	Yes

#### Radiomics models

One of the new avenues in modelling is artificial intelligence (AI) and machine learning to improve the accuracy of predictive models. AI can be used on standard clinicopathological datasets to improve the accuracy of outcome prediction. Radiomics is the method of extracting “features” related to the geometrical or physical properties of the tissue represented in a standard image using pattern-recognising algorithms. These “features” are translated into a set of numbers representing the physical properties of the lesion and may reflect its biological properties. AI can then analyse radiomics-based features extracted from standard imaging modalities to predict the outcome of the lesion under evaluation [105]. Radiomics has been applied to mammograms, tomograms, ultrasound, computerised tomography, PET, and MRI scans. Table 5 summarises the available radiomics models in sentinel node prediction. Researchers have found that this approach can predict sentinel node status more accurately than the existing clinicopathological models [106–111]. 

**Table 5:** Models using radiomics for prediction of sentinel node status classified based on the imaging modality used

USS
Lee [113]
Lee [114]
Gao [115]
Zha [116]
Yu [111]
Qiu [117]
USS and Shear Wave elastography
Jiang [118]
USS by automated breast volume scanning
Wang [119]
MMG
Tan [110]
Yang [109]
CESM
Mao [120]
PET Scan
Song [83]
Cheng [121]
CT scan
Zhang [122]
MRI
Qiu [123]
Wang [124]
Shan [125]
Wang [126]
Yu [127]
Yu [128]
Chen [129]
Song [130]
Obeid [131]
Li [132]
Liu [133]
Tan [134]
Zhang [135]
Han [107]
Zhang [136]
Mao [120]

CESM Contrast Enhanced Spectral Mammography

#### Genomic models

Current advances in molecular and genetic aspects of breast cancer have opened new insights into tumour behaviour and prognosis. Genomic data has revealed characteristic gene amplification patterns associated with different breast cancer subtypes [137, 138]. These cancer subtypes, which are not identifiable by regular histological examination, strongly relate to prognosis and tumour behaviour. Commercially available tests based on this principle, such as the “Oncotype Dx recurrence score” (validated for estimating systemic recurrence risk), have been found to predict locoregional recurrence [139]. These molecular subtypes of breast cancer can be approximately identified based on the expression of receptors (ER, PR and HER2) and the proliferation marker Ki 67 [140]. Such classification has been found to help predict tumour behaviour, including axillary lymph node metastasis. HER2 /NEU overexpression is related to the risk of axillary metastasis [141]. Progesterone receptors and S-phase fraction can significantly contribute to the prediction of nodal involvement [12]. A recent meta-analysis has found that MMP expression is associated with an increased risk of lymph node metastasis [142]. MAM, LRP, MDR1, Nup88, CXCR4, VEGF, COX2, and PIK3R5 are other genes associated with the risk of lymph node metastasis [143–151]. Low expression of the “nm 23 gene” is also associated with the risk of nodal metastasis [152, 153]. Primary tumour miRNA signature is predictive of nodal status as are alterations of tumour cell-surface glycosylation and tumour neo-angiogenesis [154–156]. A recent review by Cavalli et al. concluded that molecular markers identified on the primary tumour can potentially replace sentinel node-based assessment of the axilla [157]. Fehm et al. express a similar view. Tumour biology detected on the core biopsy of the primary tumour will help estimate a high-risk phenotype and risk of nodal involvement, hence the need for axillary dissection [73]. 

## Conclusion

Sentinel node-based management of the axilla has served well for the management of early breast cancer, and to date, alternative axillary assessment strategies have failed to produce any impact. However, SNB has potential side effects and a reported false negative rate of 6–10%, and SNB has no effect on disease-free or overall survival. Based on the available research evidence, an alternative to SNB can be developed based on clinical, radiological and genomic data. As completion axillary dissection is no longer recommended for patients with less than two nodes involved with cancer, a non-invasive model to predict the number of involved nodes could be valuable in de-escalating axillary management. It is also likely that these models, even if they don’t accurately predict nodal status in all cases, may accurately predict prognosis, will have the same value as that of the nodal status in prognostication, and hence can replace SNB in most patients with EBC.

## Data Availability

Data will not be shared as this is a descriptive review, and there is no primary data to share other than the references.

## References

[1] Cancer data in Australia. Cancer risk data visualisation - Australian Institute of Health and Welfare [Internet]. [cited 2021 Feb 12]. https://www.aihw.gov.au/reports/cancer/cancer-data-in-australia/contents/cancer-risk-data-visualisation.

[2] HALSTED WS THE RESULTS OF RADICAL OPERATIONS FOR THE CURE OF CARCINOMA OF THE BREAST.* (1907). Ann Surg.

[3] Fisher B, Jeong J-H, Anderson S, Bryant J, Fisher ER, Wolmark N (2002). Twenty-five-year follow-up of a Randomized Trial comparing Radical Mastectomy, total mastectomy, and total mastectomy followed by irradiation. New Engl J Med.

[4] Randomized Trial Comparing Axillary Clearance Versus No Axillary Clearance in Older Patients With Breast Cancer (2006). First results of international breast Cancer Study Group Trial 10–93. J Clin Oncol.

[5] Veronesi U, Orecchia R, Zurrida S, Galimberti V, Luini A, Veronesi P (2005). Avoiding axillary dissection in breast cancer surgery: a randomized trial to assess the role of axillary radiotherapy. Ann Oncol.

[6] Axelsson CK, Mouridsen HT, Zedeler K, (DBCG) DBCCG (1992). Axillary dissection of level I and II lymph nodes is important in breast cancer classification. Eur J Cancer.

[7] Fisher B, Wolmark N, Bauer M, Redmond C, Gebhardt M (1981). The accuracy of clinical nodal staging and of limited axillary dissection as a determinant of histologic nodal status in carcinoma of the breast. Surg Gynecol Obstet.

[8] Reimer T, Stachs A, Nekljudova V, Loibl S, Hartmann S, Wolter K (2017). Restricted Axillary staging in clinically and sonographically node-negative early invasive breast Cancer (c/iT1?2) in the context of breast conserving therapy: first results following commencement of the Intergroup-Sentinel-Mamma (INSEMA) Trial. Geburtsh Frauenheilk.

[9] Martelli G, Boracchi P, Ardoino I, Lozza L, Bohm S, Vetrella G (2012). Axillary Dissection Versus No Axillary dissection in older patients with T1N0 breast Cancer. Ann Surg.

[10] Agresti R, Martelli G, Sandri M, Tagliabue E, Carcangiu ML, Maugeri I (2014). Axillary lymph node dissection versus no dissection in patients with T1N0 breast cancer: a randomized clinical trial (INT09/98). Cancer.

[11] Deckers PJ (1991). Axillary dissection in breast cancer: when, why, how much, and for how long? Another operation soon to be extinct?. J Surg Oncol.

[12] Ravdin P, Laurentiis DM, Vendely T, Clark G (1994). Prediction of Axillary Lymph Node Status in breast Cancer patients by Use of Prognostic indicators. Jnci J Natl Cancer Inst.

[13] Galea MH, Blamey RW, Elston CE, Ellis IO (1992). The Nottingham prognostic index in primary breast cancer. Breast Cancer Res Tr.

[14] Goldhirsch A, Wood W, Gelber R, Coates A, Thürlimann B, Senn -JH (2007). Progress and promise: highlights of the international expert consensus on the primary therapy of early breast cancer 2007. Ann Oncol.

[15] Michaelson JS, Silverstein M, Sgroi D, Cheongsiatmoy JA, Taghian A, Powell S (2003). The effect of tumor size and lymph node status on breast carcinoma lethality. Cancer.

[16] Rosen PP, Groshen S, Saigo PE, Kinne DW, Hellman S (1989). Pathological prognostic factors in stage I (T1N0M0) and stage II (T1N1M0) breast carcinoma: a study of 644 patients with median follow-up of 18 years. J Clin Oncol.

[17] de de Bellefon M, Lemanski C, Ducteil A, Fenoglietto P, Azria D, Bourgier C (2018). Management of the Axilla in the era of breast Cancer heterogeneity. Front Oncol.

[18] de Boer M, van Dijck J, Bult P, Borm G, Tjan-Heijnen V (2010). Breast Cancer Prognosis and Occult Lymph Node Metastases, isolated Tumor cells, and Micrometastases. Jnci J Natl Cancer Inst.

[19] Reed J, Rosman M, Verbanac KM, Mannie A, Cheng Z, Tafra L (2009). Prognostic implications of isolated Tumor cells and micrometastases in Sentinel nodes of patients with invasive breast Cancer: 10-Year analysis of patients enrolled in the Prospective East Carolina University/Anne Arundel Medical Center Sentinel Node Multicenter Study. J Am Coll Surg.

[20] Fisher B, Bauer M, Wickerham DL, Redmond CK, Fisher ER, Cruz AB (1983). Relation of number of positive axillary nodes to the prognosis of patients with primary breast cancer. An NSABP update. Cancer.

[21] Nottegar A, Veronese N, Senthil M, Roumen RM, Stubbs B, Choi AH (2016). Extra-nodal extension of sentinel lymph node metastasis is a marker of poor prognosis in breast cancer patients: a systematic review and an exploratory meta-analysis. Eur J Surg Oncol Ejso.

[22] Krag DN, Weaver DL, Alex JC, Fairbank JT (1993). Surgical resection and radiolocalization of the sentinel lymph node in breast cancer using a gamma probe. Surg Oncol.

[23] Giuliano AE, Dale PS, Turner RR, Morton DL, Evans SW, Krasne DL (1995). Improved Axillary staging of breast Cancer with Sentinel Lymphadenectomy. Ann Surg.

[24] Miltenburg D, Miller C, Karamlou T, Brunicardi F (1999). Meta-analysis of sentinel lymph node biopsy in breast cancer. J Surg Res.

[25] Gill G, Centre S, of the of and (2008). Sentinel-lymph-node-based management or routine axillary clearance? One-year outcomes of sentinel node biopsy versus axillary clearance (SNAC): a randomized controlled surgical trial. Ann Surg Oncol.

[26] Straver ME, Meijnen P, van Tienhoven G, van de Velde CJ, Mansel RE, Bogaerts J (2010). Sentinel node identification rate and nodal involvement in the EORTC 10981–22023 AMAROS trial. Ann Surg Oncol.

[27] Gipponi M, Bassetti C, Canavese G, Catturich A, Somma C, Vecchio C (2004). Sentinel lymph node as a new marker for therapeutic planning in breast cancer patients. J Surg Oncol.

[28] Krag DN, Anderson SJ, Julian TB, Brown AM, Harlow SP, Costantino JP (2010). Sentinel-lymph-node resection compared with conventional axillary-lymph-node dissection in clinically node-negative patients with breast cancer: overall survival findings from the NSABP B-32 randomised phase 3 trial. Lancet Oncol.

[29] Veronesi U, Paganelli G, Viale G, Luini A. A randomized comparison of sentinel-node biopsy with routine axillary dissection in breast cancer. 2003. 10.1056/NEJMoa012782 12904519

[30] Marrazzo A, Taormina P, Gebbiab V, David M, Riili I, Gerfo DL (2007). Is sentinel lymph node biopsy more accurate than axillary dissection for staging nodal involvement in breast cancer patients?. Chir Italiana.

[31] Fleissig A, Fallowfield LJ, Langridge CI, Johnson L, Newcombe RG, Dixon JM (2006). Post-operative arm morbidity and quality of life. Results of the ALMANAC randomised trial comparing sentinel node biopsy with standard axillary treatment in the management of patients with early breast cancer. Breast Cancer Res Tr.

[32] Armer J, Fu MR, Wainstock JM, Zagar E, Jacobs LK (2004). Lymphedema following breast cancer treatment, including sentinel lymph node biopsy. Lymphology.

[33] Bromham N, Schmidt-Hansen M, Astin M, Hasler E, Reed MW (2017). Axillary treatment for operable primary breast cancer. Cochrane Libr.

[34] Peintinger F, Reitsamer R, Stranzl H, Ralph G (2003). Comparison of quality of life and arm complaints after axillary lymph node dissection vs sentinel lymph node biopsy in breast cancer patients. Brit J Cancer.

[35] Rietman JS, Dijkstra PU, Geertzen JHB, Baas P, de Vries J, Dolsma WV (2004). Treatment-related Upper Limb morbidity 1 year after Sentinel Lymph Node Biopsy or Axillary Lymph Node Dissection for Stage I or II breast Cancer. Ann Surg Oncol.

[36] Chen J, Chen J, Yang B, Liu Z, Huang X, Liu G (2012). Predicting sentinel lymph node metastasis in a Chinese breast cancer population: assessment of an existing nomogram and a new predictive nomogram. Breast Cancer Res Tr.

[37] van Roozendaal LM, Vane MLG, van Dalen T, van der Hage JA, Strobbe LJA, Boersma LJ (2017). Clinically node negative breast cancer patients undergoing breast conserving therapy, sentinel lymph node procedure versus follow-up: a Dutch randomized controlled multicentre trial (BOOG 2013-08). BMC Cancer.

[38] Krag DN, Anderson SJ, Julian TB, Brown AM, Harlow SP, Ashikaga T (2007). Technical outcomes of sentinel-lymph-node resection and conventional axillary-lymph-node dissection in patients with clinically node-negative breast cancer: results from the NSABP B-32 randomised phase III trial. Lancet Oncol.

[39] Ban E, Lee J, Koo J, Park S, Kim S, Park B-W (2011). How many Sentinel Lymph nodes are enough for Accurate Axillary staging in T1-2 breast Cancer?. J Breast Cancer.

[40] Kim MK, Park HS, Kim JY, Kim S, Nam S, Park S (2017). The clinical implication of the number of lymph nodes harvested during sentinel lymph node biopsy and its effects on survival outcome in patients with node-negative breast cancer. Am J Surg.

[41] Cipolla C, Galvano A, Vieni S, Saputo F, Lupo S, Latteri M (2021). Effects of the number of removed lymph nodes on survival outcome in patients with sentinel node-negative breast cancer. World J Surg Oncol.

[42] Bernardi S, Bertozzi S, Londero AP, Angione V, Petri R, Giacomuzzi F (2013). Prevalence and risk factors of intraoperative identification failure of sentinel lymph nodes in patients affected by breast cancer. Nucl Med Commun.

[43] Cady B, Stone MD, Schuler JG, Thakur R, Wanner MA, Lavin PT (1996). The New era in breast Cancer: Invasion, size, and nodal involvement dramatically decreasing as a result of Mammographic Screening. Arch Surg-chicago.

[44] Tabár L, Duffy SW, Vitak B, Chen H, Prevost TC (1999). The natural history of breast carcinoma. Cancer.

[45] Jatoi I, Benson JR, Toi M (2016). De-escalation of axillary surgery in early breast cancer. Lancet Oncol.

[46] Veronesi U, Paganelli G, Viale G, Luini A, Zurrida S, Galimberti V (2006). Sentinel-lymph-node biopsy as a staging procedure in breast cancer: update of a randomised controlled study. Lancet Oncol.

[47] Voogd AC, Coebergh J-WW, van Driel OJR, Roumen RMH, van Beek MWPM, Vreugdenhil A (2000). The risk of nodal metastases in breast cancer patients with clinically negative lymph nodes: a population-based analysis. Breast Cancer Res Tr.

[48] Kamath VJ, Giuliano R, Dauway EL, Cantor A, Berman C, Ku NN (2001). Characteristics of the Sentinel Lymph Node in breast Cancer predict further involvement of Higher-Echelon Nodes in the Axilla: a study to evaluate the need for complete Axillary Lymph Node Dissection. Arch Surg-chicago.

[49] Rahusen FD, Torrenga H, van Diest PJ, Pijpers R, van der Wall E, Licht J (2001). Predictive factors for metastatic involvement of Nonsentinel nodes in patients with breast Cancer. Arch Surg-chicago.

[50] Giuliano AE, Hunt KK, Ballman KV, Beitsch PD, Whitworth PW, Blumencranz PW (2011). Axillary dissection vs no axillary dissection in women with invasive breast cancer and sentinel node metastasis: a randomized clinical trial. JAMA.

[51] Galimberti V, Cole BF, Zurrida S, Viale G, Luini A, Veronesi P (2013). Axillary dissection versus no axillary dissection in patients with sentinel-node micrometastases (IBCSG 23 – 01): a phase 3 randomised controlled trial. Lancet Oncol.

[52] Donker M, Tienhoven G, Straver ME, Meijnen P, Velde CJ, Mansel RE (2014). Radiotherapy or surgery of the axilla after a positive sentinel node in breast cancer (EORTC 10981–22023 AMAROS): a randomised, multicentre, open-label, phase 3 non-inferiority trial. Lancet Oncol.

[53] Fu Y, Chung D, Cao M-A, Apple S, Chang H (2014). Is Axillary Lymph Node Dissection necessary after Sentinel Lymph Node Biopsy in patients with mastectomy and pathological N1 breast Cancer?. Ann Surg Oncol.

[54] Sávolt Á, Péley G, Polgár C, Udvarhelyi N, Rubovszky G, Kovács E (2017). Eight-year follow up result of the OTOASOR trial: the optimal treatment of the axilla – surgery or Radiotherapy after positive sentinel lymph node biopsy in early-stage breast cancer A randomized, single centre, phase III, non-inferiority trial. Eur J Surg Oncol Ejso.

[55] Rutgers EJ, Donker M, Straver ME, Meijnen P, Velde CJHVD, Mansel RE (2013). Radiotherapy or surgery of the axilla after a positive sentinel node in breast cancer patients: final analysis of the EORTC AMAROS trial (10981/22023). J Clin Oncol.

[56] Giuliano AE, Ballman KV, McCall L, Beitsch PD, Brennan MB, Kelemen PR (2017). Effect of Axillary Dissection vs no Axillary dissection on 10-Year overall survival among women with invasive breast Cancer and Sentinel Node Metastasis: the ACOSOG Z0011 (Alliance) Randomized Clinical Trial. JAMA.

[57] Galimberti V, Cole BF, Viale G, Veronesi P, Vicini E, Intra M (2018). Axillary dissection versus no axillary dissection in patients with breast cancer and sentinel-node micrometastases (IBCSG 23 – 01): 10-year follow-up of a randomised, controlled phase 3 trial. Lancet Oncol.

[58] Giuliano AE, McCall L, Beitsch P, Whitworth PW, Blumencranz P, Leitch MA et al. Locoregional recurrence after sentinel lymph node dissection with or without axillary dissection in patients with sentinel lymph node metastases: the American College of Surgeons Oncology Group Z0011 randomized trial. Ann Surg. 2010. 10.1097/SLA.0b013e3181f08f32 PMC5593421 20739842

[59] Castelo M, Hu SY, Dossa F, Acuna SA, Scheer AS (2020). Comparing Observation, Axillary Radiotherapy, and Completion Axillary Lymph Node Dissection for Management of Axilla in breast Cancer in patients with positive Sentinel nodes: a systematic review. Ann Surg Oncol.

[60] Crawford JD, Ansteth M, Barnett J, Glissmeyer M, Johnson NG (2013). Routine completion axillary lymph node dissection for positive sentinel nodes in patients undergoing mastectomy is not associated with improved local control. Am J Surg.

[61] Cady B (1997). Case against axillary lymphadenectomy for most patients with infiltrating breast cancer. J Surg Oncol.

[62] Chua B, Ung O, Taylor R, Boyages J (2001). Frequency and predictors of axillary lymph node metastases in invasive breast cancer. Anz J Surg.

[63] Ditsch N, Rubio IT, Gasparri ML, de Boniface J, Kuehn T (2020). Breast and axillary surgery in malignant breast disease: a review focused on literature of 2018 and 2019. Curr Opin Obstet Gyn.

[64] Soni NK, Carmalt HL, Gillett DJ, Spillane AJ (2005). Evaluation of a breast cancer nomogram for prediction of non-sentinel lymph node positivity. Eur J Surg Oncol Ejso.

[65] Viale G, Zurrida S, Maiorano E, Mazzarol G, Pruneri G, Paganelli G (2005). Predicting the status of axillary sentinel lymph nodes in 4351 patients with invasive breast carcinoma treated in a single institution. Cancer.

[66] Fisher B, Dignam J, Tan-Chiu E, Anderson S, Fisher ER, Wittliff JL (2001). Prognosis and treatment of patients with breast tumors of one centimeter or less and negative Axillary Lymph Nodes. Jnci J Natl Cancer Inst.

[67] Lippman ME, Hayes DF (2001). Adjuvant therapy for all patients with breast Cancer?. Jnci J Natl Cancer Inst.

[68] Goldhirsch A, Ingle JN, Gelber RD, Coates AS, Thürlimann B, Senn H-J (2009). Thresholds for therapies: highlights of the St Gallen International Expert Consensus on the primary therapy of early breast Cancer 2009. Ann Oncol.

[69] Cady B (1994). The need to reexamine axillary lymph node dissection in invasive breast cancer. Cancer.

[70] Straver ME, Meijnen P, van Tienhoven G, van de Velde CJH, Mansel RE, Bogaerts J (2009). Role of Axillary Clearance after a tumor-positive Sentinel Node in the administration of adjuvant therapy in early breast Cancer. J Clin Oncol.

[71] Gentilini O, Veronesi U (2012). Abandoning sentinel lymph node biopsy in early breast cancer? A new trial in progress at the European Institute of Oncology of Milan (SOUND: Sentinel node vs Observation after axillary UltraSouND). Breast.

[72] Martelli G, Miceli R, Daidone MG, Vetrella G, Cerrotta AM, Piromalli D (2011). Axillary Dissection Versus No Axillary Dissection in Elderly patients with breast Cancer and no palpable Axillary nodes: results after 15 years of Follow-Up. Ann Surg Oncol.

[73] Fehm T, Maul H, Gebauer S, Scharf A, Baier P, Sohn C (2005). Prediction of Axillary Lymph Node Status of breast Cancer patients by tumorbiological factors of the primary tumor. Strahlenther Onkol.

[74] Lyman GH, Somerfield MR, Bosserman LD, Perkins CL, Weaver DL, Giuliano AE (2016). Sentinel Lymph Node Biopsy for patients with early-stage breast Cancer: American Society of Clinical Oncology Clinical Practice Guideline Update. J Clin Oncol.

[75] Tucker NS, Gillanders WE, Eberlein T, Aft R, Margenthaler J, Gao F et al. Abstract OT3-5-01: A prospective, randomized trial of sentinel lymph node biopsy versus no additional staging in patients with T1-T2 invasive breast cancer and negative axillary ultrasound. Ongoing Clin Trials. 2015;OT3-5-01-OT3-5-01.

[76] Brackstone M, Baldassarre FG, Perera FE, Cil T, Gregor MCM, Dayes IS (2021). Management of the Axilla in early-stage breast Cancer: Ontario Health (Cancer Care Ontario) and ASCO Guideline. J Clin Oncol.

[77] Gentilini OD, Botteri E, Sangalli C, Galimberti V, Porpiglia M, Agresti R (2023). Sentinel Lymph Node Biopsy vs no axillary surgery in patients with small breast Cancer and negative results on Ultrasonography of Axillary Lymph Nodes. JAMA Oncol.

[78] Farrell TPJ, Adams NC, Stenson M, Carroll PA, Griffin M, Connolly EM (2015). The Z0011 trial: is this the end of axillary ultrasound in the pre-operative assessment of breast cancer patients?. Eur Radiol.

[79] Lee B, Lim AK, Krell J, Satchithananda K, Coombes RC, Lewis JS (2013). The efficacy of Axillary Ultrasound in the detection of nodal metastasis in breast Cancer. Am J Roentgenol.

[80] Moody AN, Bull J, Culpan A-M, Munyombwe T, Sharma N, Whitaker M (2017). Preoperative sentinel lymph node identification, biopsy and localisation using contrast enhanced ultrasound (CEUS) in patients with breast cancer: a systematic review and meta-analysis. Clin Radiol.

[81] Li J, Wang S-R, Li Q-L, Zhu T, Zhu P-S, Chen M (2023). Diagnostic value of multiple ultrasound diagnostic techniques for axillary lymph node metastases in breast cancer: a systematic analysis and network meta-analysis. Front Oncol.

[82] Kuijs V, Moossdorff M, Schipper R, Beets-Tan R, Heuts E, Keymeulen K (2015). The role of MRI in axillary lymph node imaging in breast cancer patients: a systematic review. Insights into Imaging.

[83] Song B-I (2021). A machine learning-based radiomics model for the prediction of axillary lymph-node metastasis in breast cancer. Breast Cancer-tokyo.

[84] Bevilacqua J, Kattan M, Fey J. Doctor, what are my chances of having a positive sentinel node? A validated nomogram for risk estimation. 2007. 10.1200/JCO.2006.08.8013 17664461

[85] Carmichael AR, Aparanji K, Nightingale P, Boparai R, Stonelake PS (2006). A clinicopathological scoring system to select breast cancer patients for sentinel node biopsy. Eur J Surg Oncol Ejso.

[86] Chen K, Liu J, Li S, Jacobs L (2017). Development of nomograms to predict axillary lymph node status in breast cancer patients. BMC Cancer.

[87] Reyal F, Rouzier R, Depont-Hazelzet B, Bollet MA, Pierga J-Y, Alran S et al. The Molecular Subtype classification is a determinant of Sentinel Node Positivity in early breast carcinoma. PLoS ONE. 2011;6. 10.1371/journal.pone.0020297 PMC3105053 21655258

[88] KOLARIK D, PECHA V, SKOVAJSOVA M, ZAHUMENSKY J, TRNKOVA M (2013). Predicting axillary sentinel node status in patients with primary breast cancer. Neoplasma.

[89] Xie F, Yang H, Wang S, Zhou B, Tong F, Yang D (2012). A logistic regression model for Predicting Axillary Lymph Node metastases in early breast carcinoma patients. Sensors.

[90] Qiu S-Q, Zeng H-C, Zhang F, Chen C, Huang W-H, Pleijhuis RG (2016). A nomogram to predict the probability of axillary lymph node metastasis in early breast cancer patients with positive axillary ultrasound. Sci Rep-uk.

[91] Chen W, Wang C, Fu F, Yang B, Chen C, Sun Y (2020). A model to predict the risk of Lymph Node Metastasis in breast Cancer based on clinicopathological characteristics. Cancer Manage Res.

[92] Silverstein MJ, Skinner KA, Lomis TJ (2001). Predicting Axillary nodal positivity in 2282 patients with breast carcinoma. World J Surg.

[93] Takada M, Sugimoto M, Naito Y, Moon H-G, Han W, Noh D-Y (2012). Prediction of axillary lymph node metastasis in primary breast cancer patients using a decision tree-based model. Bmc Med Inf Decis.

[94] Olivotto IA, Jackson JSH, Mates D, Andersen S, Davidson W, Bryce CJ (1998). Prediction of axillary lymph node involvement of women with invasive breast carcinoma. Cancer.

[95] Gann PH, Colilla SA, Gapstur SM, Winchester DJ, Winchester DP (1999). Factors associated with axillary lymph node metastasis from breast carcinoma. Cancer.

[96] Greer LT, Rosman M, Mylander CW, Liang W, Buras RR, Chagpar AB et al. A prediction model for the Presence of Axillary Lymph Node involvement in women with invasive breast Cancer: a Focus on Older Women. Breast J. 2014;20. 10.1111/tbj.12233 24475876

[97] Chagpar AB, McMasters KM, Edwards MJ (2010). Can Sentinel Node Biopsy be avoided in some Elderly breast Cancer patients?. Ann Surg [Internet].

[98] Chadha M, Chabon AB, Friedmann P, Vikram B (1994). Predictors of axillary lymph node metastases in patients with T1 breast cancer. A multivariate analysis. Cancer.

[99] Ahlgren J, Westman G, Stål O, Arnesson L-G (2009). Prediction of Axillary Lymph Node metastases in a screened breast Cancer Population. Acta Oncol.

[100] Laurentiis MD, Gallo C, Placido SD, Perrone F, Pettinato G, Petrella G (1996). A predictive index of axillary nodal involvement in operable breast cancer. Brit J Cancer.

[101] Barth A, Craig PH, Silverstein MJ (1997). Predictors of axillary lymph node metastases in patients with T1 breast carcinoma. Cancer.

[102] Choong PL, deSilva CJS, Dawkins HJS, Sterrett GF, Robbins P, Harvey JM (1996). Predicting axillary lymph node metastases in breast carcinoma patients. Breast Cancer Res Tr.

[103] Mustafa IA, Cole B, Wanebo HJ, Bland KI, Chang HR (1997). The impact of histopathology on nodal metastases in minimal breast Cancer. Arch Surg-chicago.

[104] Patani N, Dwek M, Douek M (2007). Predictors of axillary lymph node metastasis in breast cancer: a systematic review. Eur J Surg Oncology: J Eur Soc Surg Oncol Br Association Surg Oncol.

[105] Tagliafico AS, Piana M, Schenone D, Lai R, Massone AM, Houssami N (2020). Overview of radiomics in breast cancer diagnosis and prognostication. Breast.

[106] Dong Y, Feng Q, Yang W, Lu Z, Deng C, Zhang L (2018). Preoperative prediction of sentinel lymph node metastasis in breast cancer based on radiomics of T2-weighted fat-suppression and diffusion-weighted MRI. Eur Radiol.

[107] Han L, Zhu Y, Liu Z, Yu T, He C, Jiang W (2019). Radiomic nomogram for prediction of axillary lymph node metastasis in breast cancer. Eur Radiol.

[108] Mao N, Dai Y, Lin F, Ma H, Duan S, Xie H (2020). Radiomics Nomogram of DCE-MRI for the prediction of Axillary Lymph Node Metastasis in breast Cancer. Front Oncol.

[109] Yang J, Wang T, Yang L, Wang Y, Li H, Zhou X (2019). Preoperative prediction of Axillary Lymph Node Metastasis in breast Cancer using mammography-based Radiomics Method. Sci Rep-uk.

[110] Tan H, Wu Y, Bao F, Zhou J, Wan J, Tian J (2020). Mammography-based radiomics nomogram: a potential biomarker to predict axillary lymph node metastasis in breast cancer. Br J Radiol.

[111] Yu F-H, Wang J-X, Ye X-H, Deng J, Hang J, Yang B (2019). Ultrasound-based radiomics nomogram: a potential biomarker to predict axillary lymph node metastasis in early-stage invasive breast cancer. Eur J Radiol.

[112] Breast Cancer Nomogram to Predict Positive Sentinel Lymph. Nodes, without Neoadjuvant Chemotherapy [Internet]. [cited 2023 Sep 14]. https://www3.mdanderson.org/app/medcalc/bc_nomogram3/index.cfm?pagename=sln.

[113] Lee Y-W, Huang C-S, Shih C-C, Chang R-F (2021). Axillary lymph node metastasis status prediction of early-stage breast cancer using convolutional neural networks. Comput Biol Med.

[114] Lee SE, Sim Y, Kim S, Kim E-K (2020). Feasibility of ultrasonography-based radiomics to predict axillary lymph node metastasis in the preoperative evaluation of breast cancer. Ultrasonography.

[115] Gao Y, Luo Y, Zhao C, Xiao M, Ma L, Li W (2021). Nomogram based on radiomics analysis of primary breast cancer ultrasound images: prediction of axillary lymph node tumor burden in patients. Eur Radiol.

[116] Zha H, Zong M, Liu X, Pan J, Wang H, Gong H (2021). Preoperative ultrasound-based radiomics score can improve the accuracy of the Memorial Sloan Kettering Cancer Center nomogram for predicting sentinel lymph node metastasis in breast cancer. Eur J Radiol.

[117] Qiu X, Jiang Y, Zhao Q, Yan C, Huang M, Jiang T (2020). Could Ultrasound-based Radiomics noninvasively predict axillary lymph node metastasis in breast Cancer?. J Ultras Med.

[118] Jiang M, Li C-L, Luo X-M, Chuan Z-R, Chen R-X, Tang S-C (2022). Radiomics model based on shear-wave elastography in the assessment of axillary lymph node status in early-stage breast cancer. Eur Radiol.

[119] Wang H, Yang X, Chen F, Qin Y, Li X, Ma S (2023). Non-invasive Assessment of Axillary Lymph Node Metastasis risk in early invasive breast Cancer adopting automated breast volume scanning-based Radiomics Nomogram: a Multicenter Study. Ultrasound Med Biology.

[120] Mao N, Yin P, Li Q, Wang Q, Liu M, Ma H (2020). Radiomics nomogram of contrast-enhanced spectral mammography for prediction of axillary lymph node metastasis in breast cancer: a multicenter study. Eur Radiol.

[121] Cheng J, Ren C, Liu G, Shui R, Zhang Y, Li J (2022). Development of high-resolution dedicated PET-Based Radiomics Machine Learning Model to Predict Axillary Lymph Node Status in early-stage breast Cancer. Cancers.

[122] Zhang J, Cao G, Pang H, Li J, Yao X (2022). Development and validation of radiomics machine learning model based on contrast-enhanced computed tomography to predict axillary lymph node metastasis in breast cancer. Bosnian J Basic Med.

[123] Qiu X, Fu Y, Ye Y, Wang Z, Cao C (2022). A Nomogram based on molecular biomarkers and Radiomics to predict lymph node metastasis in breast Cancer. Front Oncol.

[124] Wang D, Hu Y, Zhan C, Zhang Q, Wu Y, Ai T (2022). A nomogram based on radiomics signature and deep-learning signature for preoperative prediction of axillary lymph node metastasis in breast cancer. Front Oncol.

[125] Shan Y, Xu W, Wang R, Wang W, Pang P, Shen Q (2020). A Nomogram Combined Radiomics and kinetic curve pattern as Imaging Biomarker for detecting metastatic axillary lymph node in invasive breast Cancer. Front Oncol.

[126] Wang C, Chen X, Luo H, Liu Y, Meng R, Wang M (2021). Development and Internal Validation of a preoperative prediction model for Sentinel Lymph Node Status in breast Cancer: combining Radiomics signature and clinical factors. Front Oncol.

[127] Yu Y, Tan Y, Hu Q, Ouyang J, Chen Y, Yang G (2020). Development and validation of a magnetic resonance imaging radiomics-based signature to predict axillary lymph node metastasis and disease-free survival in patients with breast cancer: a multicenter cohort study. Ann Oncol.

[128] Yu Y, Tan Y, Xie C, Hu Q, Ouyang J, Chen Y (2020). Development and validation of a preoperative magnetic resonance imaging Radiomics–Based signature to Predict Axillary Lymph Node Metastasis and Disease-Free Survival in patients with early-stage breast Cancer. Jama Netw Open.

[129] Chen H, Lan X, Yu T, Li L, Tang S, Liu S (2022). Development and validation of a radiogenomics model to predict axillary lymph node metastasis in breast cancer integrating MRI with transcriptome data: a multicohort study. Front Oncol.

[130] Song D, Yang F, Zhang Y, Guo Y, Qu Y, Zhang X (2022). Dynamic contrast-enhanced MRI radiomics nomogram for predicting axillary lymph node metastasis in breast cancer. Cancer Imaging.

[131] Obeid JP, Stoyanova R, Zeidan Y (2017). Multiparametric breast MRI analysis predicts Axillary nodal involvement in early stage breast Cancer. Int J Radiat Oncol Biology Phys.

[132] Li L, Yu T, Sun J, Jiang S, Liu D, Wang X (2021). Prediction of the number of metastatic axillary lymph nodes in breast cancer by radiomic signature based on dynamic contrast-enhanced MRI. Acta Radiol.

[133] Liu Y, Li X, Zhu L, Zhao Z, Wang T, Zhang X (2022). Preoperative prediction of Axillary Lymph Node Metastasis in breast Cancer based on Intratumoral and Peritumoral DCE-MRI Radiomics Nomogram. Contrast Media Mol I.

[134] Tan H, Gan F, Wu Y, Zhou J, Tian J, Lin Y (2020). Preoperative prediction of Axillary Lymph Node Metastasis in breast carcinoma using Radiomics features based on the Fat-suppressed T2 sequence. Acad Radiol.

[135] Zhang X, Yang Z, Cui W, Zheng C, Li H, Li Y (2021). Preoperative prediction of axillary sentinel lymph node burden with multiparametric MRI-based radiomics nomogram in early-stage breast cancer. Eur Radiol.

[136] Zhang J, Zhang Z, Mao N, Zhang H, Gao J, Wang B (2023). Radiomics nomogram for predicting axillary lymph node metastasis in breast cancer based on DCE-MRI: a multicenter study. Xst.

[137] Perou CM, Sørlie T, Eisen MB, Rijn M, van de, Jeffrey SS, Rees CA (2000). Molecular portraits of human breast tumours. Nature.

[138] Sørlie T, Perou C, Tibshirani R. Gene expression patterns of breast carcinomas distinguish tumor subclasses with clinical implications. 2001. 10.1073/pnas.191367098 PMC58566 11553815

[139] Mamounas EP, Liu Q, Paik S, Baehner FL, Tang G, Jeong J-H (2017). 21-Gene recurrence score and Locoregional recurrence in Node-Positive/ER-Positive breast Cancer treated with chemo-endocrine therapy. J Natl Cancer I.

[140] Goldhirsch A, Winer E, Coates A, Gelber R, Piccart-Gebhart M, Thürlimann B et al. Personalizing the treatment of women with early breast cancer: highlights of the St Gallen International Expert Consensus on the primary therapy of early breast Cancer 2013. Ann Oncol. 2013;24. 10.1093/annonc/mdt303 PMC3755334 23917950

[141] Nathanson DS, Slater R, DeBruyn D, Kapke A, Linden M (2006). HER-2/neu expression in primary breast Cancer with Sentinel Lymph Node Metastasis. Ann Surg Oncol.

[142] Chen Y, Wang X, Chen G, Dong C, Zhang D (2015). The impact of matrix metalloproteinase 2 on prognosis and clinicopathology of breast cancer patients: a systematic meta-analysis. PLoS ONE.

[143] Núñez-Villar M, Martínez-Arribas F, Pollán M, Lucas A, Sánchez J, Tejerina A (2003). Elevated mammaglobin (h-MAM) expression in breast cancer is associated with clinical and biological features defining a less aggressive tumour phenotype. Breast cancer Research: BCR.

[144] Schneider J, Gonzalez-Roces S, Pollán M, Lucas R, Tejerina A, Martin M (2001). Expression of LRP and MDR1 in locally advanced breast cancer predicts axillary node invasion at the time of rescue mastectomy after induction chemotherapy. Breast cancer Research: BCR.

[145] Agudo D, Gómez-Esquer F, Martínez-Arribas F, Núñez-Villar MJ, Pollán M, Schneider J (2004). Nup88 mRNA overexpression is associated with high aggressiveness of breast cancer. Int J cancer J Int Du cancer.

[146] Bahhnassy A, Mohanad M, Shaarawy S, Ismail MF, El-Bastawisy A, Ashmawy AM (2015). Transforming growth factor-β, insulin-like growth factor I/insulin-like growth factor I receptor and vascular endothelial growth factor-A: prognostic and predictive markers in triple-negative and non-triple-negative breast cancer. Mol Med Rep.

[147] Zhu L, Loo WT, Louis W (2007). PTEN and VEGF: possible predictors for sentinel lymph node micro-metastasis in breast cancer. Biomed Pharmacotherapy = Biomédecine pharmacothérapie.

[148] Hao L, Zhang C, Qiu Y, Wang L, Luo Y, Jin M (2007). Recombination of CXCR4, VEGF, and MMP-9 predicting lymph node metastasis in human breast cancer. Cancer Lett.

[149] Generali D, Buffa F, Deb S, Cummings M, Reid L, Taylor M (2014). COX-2 expression is predictive for early relapse and aromatase inhibitor resistance in patients with ductal carcinoma in situ of the breast, and is a target for treatment. Br J Cancer.

[150] Cubukcu E, Kanat O, Olmez FO, Kabul S, Canhoroz M, Avci N (2013). Prognostic significance of estrogen receptor, progesterone receptor, HER2/neu, Ki-67, and nm23 expression in patients with invasive breast cancer. J BUON: Official J Balkan Union Oncol.

[151] Paula LM, Moraes LHFD, Canto ALD, Santos LD, Martin AA, Rogatto SR (2017). Analysis of molecular markers as predictive factors of lymph node involvement in breast carcinoma. Oncol Lett.

[152] Hennessy C, Henry JA, May FEB, Westley BR, Angus B, Lennard TWJ. Expression of the Antimetastatic Gene nm23 in Human Breast Cancer: An Association WIth Good Prognosis. JNCI: J Natl Cancer Inst. 1991;83:281–5. 10.1093/jnci/83.4.281 1994057

[153] Royds JA, Rees RC, Stephenson TJ (1994). nm23—A metastasis suppressor gene?. J Pathol.

[154] Mumprecht V, Detmar M (2009). Lymphangiogenesis and cancer metastasis. J Cell Mol Med.

[155] Xie X, Tan W, Chen B, Huang X, Peng C, Yan S (2018). Preoperative prediction nomogram based on primary tumor miRNAs signature and clinical-related features for axillary lymph node metastasis in early‐stage invasive breast cancer. Int J Cancer.

[156] Christiansen MN, Chik J, Lee L, Anugraham M, Abrahams JL, Packer NH (2014). Cell surface protein glycosylation in cancer. Proteomics.

[157] Cavalli LR (2009). Molecular markers of breast axillary lymph node metastasis. Expert Rev Mol Diagn.

